# Prevalence and Characteristics of Plasmid-Mediated Fosfomycin Resistance Gene *fosA3* among *Salmonella* Enteritidis Isolates from Retail Chickens and Children with Gastroenteritis in China

**DOI:** 10.3390/pathogens13090816

**Published:** 2024-09-21

**Authors:** Liyuan Liu, Shanrong Yi, Xuebin Xu, Liya Zheng, Hong Liu, Xiujuan Zhou

**Affiliations:** 1 College of Public Health, Shanghai University of Medicine and Health Sciences, Shanghai 201318, China; 2 School of Agriculture and Biology, Shanghai Jiao Tong University, Shanghai 200240, China; 3 Shanghai Center for Disease Control and Prevention, Shanghai 200336, China

**Keywords:** *Salmonella* Enteritidis, *fosA3*, S1-PFGE, plasmid dissemination, molecular epidemiology

## Abstract

A total of 265 *Salmonella* Enteritidis isolates collected from retail markets and children’s hospitals in Shanghai were used to investigate the prevalence and molecular epidemiology of plasmid-mediated fosfomycin resistance genes. Nine of the isolates—7 from the 146 (4.79%) retail chicken-related samples and 2 from the 119 (1.68%) samples from clinical children—were fosfomycin-resistant (Fos^R^). The *fosA3* gene was detected in all of the nine Fos^R^ isolates, which were located on Inc F-type (8/9, 88.9%) and unknown-type (1/9, 11.1%) transferable plasmids. In total, five plasmid types, namely Inc HI2 (1/9, 11.1%), Inc I1 (3/9, 33.3%), Inc X (8/9, 88.9%), Inc FIIs (9/9, 100%), and Inc FIB (9/9, 100%), were detected in these Fos^R^ isolates, which possessed five S1 nuclease pulsed-field gel electrophoresis (S1-PFGE) profiles. The extended-spectrum β-lactamase determinant *bla*_CTX-M-14_ subtype was identified in one Fos^R^ *S.* Enteritidis isolate, which was located in a transferable unknown-type plasmid co-carrying *fosA3* and *tetR* genes. Sequence homology analysis showed that this plasmid possessed high sequence similarity to previously reported *bla*_CTX-M-14_- and *fosA3*-positive plasmids from *E. coli* strains, implying that plasmids carrying the *fosA3* gene might be disseminated among Enterobacterales. These findings highlight further challenges in the prevention and treatment of Enterobacteriaceae infections caused by plasmids containing *fosA3*.

## 1. Introduction

Because of the increase in the incidence of multidrug-resistant (MDR) bacteria and the depletion of newly developed antibiotics, the reassessment of “older” antibiotics has emerged as an interesting option. Fosfomycin was discovered five decades ago and is a promising candidate for the treatment of various MDR pathogens [1], especially extended-spectrum beta-lactamase (ESBL)-producing Enterobacterales [2,3]. However, previous studies have demonstrated that overall fosfomycin resistance in China is higher than in other parts of the world in both human and animal hosts [4]. Preliminary evidence suggests that *fosA3* is the primary gene responsible for fosfomycin resistance in common clinical pathogens in China [4], such as *Escherichia coli* and *Klebsiella pneumoniae* isolates [5,6,7], spread through the dissemination of Inc F and Inc N plasmids rather than the clonal expansion of specific strains [8,9]. Therefore, these plasmids may be spread among enterobacteria, including foodborne pathogens.

Foodborne salmonellosis has become a serious public health issue worldwide. *Salmonella* is a problematic bacterial pathogen that is frequently resistant to multiple antibiotics. Although the prevalence rate of fosfomycin resistance in *Salmonella* remains unclear, the gene *fosA3* has been recently found among fosfomycin-resistant *Salmonella* isolates from wild birds [10], food animals [11,12,13,14], and hospitalized patients [15,16,17]; it was also found in isolates obtained from food [18] and a healthy catering worker [19]. Further analysis showed that the genetic environment of the *fosA3* gene in *Salmonella* had a structure identical to that in *E. coli* isolates, indicating that the transferability of *fosA3*-harboring plasmids in enterobacteria accounts for the further transmission of antimicrobial resistance.

*Salmonella enterica* subsp. *enterica* serovar Enteritidis (*S.* Enteritidis) is the most common serovar identified in chicken meat [20] and is frequently associated with human illness [21]. Information on the occurrence and epidemiological characteristics of fosfomycin-resistant *S.* Enteritidis isolates in China remains scarce. In this study, we investigated the prevalence and molecular epidemiology of plasmid-mediated fosfomycin resistance genes in *S.* Enteritidis isolates collected from retail chicken samples and children with gastroenteritis. The transfer mechanism of fosfomycin resistance in these isolates was characterized using plasmid assays and sequencing.

## 2. Materials and Methods

### 2.1. S. Enteritidis Isolates and Fosfomycin Susceptibility Testing

*S.* Enteritidis isolates (n = 265) for this study were obtained from 146 retail chicken-related samples and 119 children under the age of 10 in Shanghai, China from 2010 to 2012. Food sources, patient demographics, and isolation dates of these isolates were reported in our previous studies [22,23]. The minimal inhibitory concentration (MIC) of the isolates against fosfomycin (FOS; Sigma, CA, USA) was determined using an agar dilution method according to the guidelines recommended by the Clinical and Laboratory Standards Institute [24]. The breakpoint for fosfomycin was MIC ≥ 256 μg/mL, according to the interpretive standards of the CLSI [24]. The following 12 antibiotics were tested in fosfomycin resistance (Fos^R^) isolates: tetracycline (TET), sulfisoxazole (SUL), ampicillin (AMP), ceftriaxone (CRO), streptomycin (STR), azithromycin (AZI), trimethoprim-sulfonamides (SXT), amikacin (AMK), ciprofloxacin (CIP), chloramphenicol (CHL), kanamycin (KAN), and gentamicin (GEN). The sensitivity analysis of the aforementioned antibiotics was evaluated using MIC, based on the disk diffusion test. Antibiotics were purchased from Sigma-Aldrich (St. Louis, MO, USA). *E. coli* ATCC 25922 and *Enterococcus faecalis* ATCC 29212 were used as quality control organisms.

### 2.2. Plasmid Studies

Plasmid incompatibility (Inc.) groups were assigned by PCR-based replicon typing using the genomic DNA of the Fos^R^ isolates as a template. Amplification by PCR was performed with 18 specific primer pairs designed for FIA, FIB, FIC, HI1, HI2, I1, L/M, N, P, W, T, A/C, K, B/O, X, Y, F, and FIIs basic replicons using a previously described corresponding protocol [25]. The presence of plasmid-encoded fosfomycin resistance (*fosA3*, *fosC2,* and *fosKP96*), β-lactams (*bla*_TEM_, *bla*_CTX-M_, and *bla*_OXA_), tetracycline (*tetA* and *tet B*), and sulfonamides (*sul1* and *sul2*) were determined by PCR as described previously by Sato et al. [26] and Zhou et al. [22]. All the primers used are listed in Tables S1 and S2. The PCR cycling parameters were as follows: initial denaturation at 94 °C for 10 min; denaturation at 94 °C for 1 min; corresponding annealing temperature conditions (Tables S1 and S2) for 30 s; extension at 72 °C for 1 min and 30 cycles; and extension at 72 °C for 10 min.

Conjugation experiments were performed using *E. coli* C600 as the recipient [27]. Briefly, each Fos^R^ *S.* Enteritidis isolate as a donor and *E. coli* C600 after overnight incubation were mixed and then transferred to a filter in Luria–Bertani broth (Oxoid, Cambridge, UK) agar plates for overnight culture. Trans-conjugants were selected on MacConkey agar plates supplemented with fosfomycin (256 μg/mL) and rifampin (200 µg/mL). Plasmids of the parental isolates and trans-conjugants were sized by S1 nuclease pulsed-field gel electrophoresis (S1-PFGE) [28].

### 2.3. Plasmid Sequencing and Analysis

Genomic DNA was extracted from overnight cultures of *S.* Enteritidis SJTUF11561 using the QIAamp DNA Mini Kit (Qiagen, CA, USA). The whole genome sequencing (WGS) was performed on the PacBio RS II sequencing platform at the Majorbio Corporation (Shanghai, China). Briefly, a 10 kb DNA library was constructed and sequenced using single-molecule real-time sequencing. The sequence data were assembled using the Canu V1.3 software [29], and the complete sequence was annotated using the RAST, BLASTn, and BLASTp programs on the NCBI platform, followed by manual inspection. Insertion sequences and repetitive elements were identified using IS finder2. A schematic plasmid map was constructed using WinPlas2.7 software. OriTfinder (http://202.120.12.134/oriTfinder/oriTfinder.html, accessed on 12 March 2024) and Plasmidfinder (https://cge.cbs.dtu.dk/services/PlasmidFinder/, accessed on 12 March 2024) were used to identify the origins of transfers and plasmid types in the DNA sequences of bacterial plasmids, respectively.

### 2.4. Nucleotide Sequence Accession Number

The complete sequences of *S.* Enteritidis SJTUF11561 and its plasmids (p11561A, p115561B, p11561C, p11561D, and p11561E) were deposited in the NCBI database under PRJNA1136444.

## 3. Results

### 3.1. Prevalence of Fosfomycin Resistance and Plasmid-Mediated Determinants

In total, 9 out of 256 (3.51%) isolates, including 7 out of 146 (4.79%) from retail chicken-related samples and 2 out of 119 (1.68%) from children with gastroenteritis, were fosfomycin-resistant (Table 1). The MIC value of two retail chicken-related isolates was 512 μg/mL; the other fosfomycin-resistant isolates had an MIC value of 256 μg/mL (Table 1 and Table 2). All Fos^R^ isolates were positive for *fosA3* (Table 2), and none possessed *fosKP96* and *fosC2* genes. The incidence of *sul2* was 100%, and *bla*_TEM_ was present in six out of nine (66.7%) Fos^R^ isolates. Two Fos^R^ isolates from chicken manure and both the isolates from clinical children carried *tetA* (four out of nine, 44.4%). Only one Fos^R^ isolate (SJTUF 11561) carried *bla*_CTX-M_ (Table 2).

### 3.2. Characteristics of FosA3-Carrying S. Enteritidis Isolates and Plasmids

All of the nine Fos^R^ isolates were resistant to two or more drugs (Table 2). The incidence of sulfisoxazole (SUL) and fosfomycin (FOS) was 100% in the Fos^R^ isolates. Specifically, strain SJTUF 11346 only possessed SUL-FOS. Except for SJTUF 11346, all of the Fos^R^ isolates from chicken-related samples possessed ampicillin (AMP), streptomycin (STR), and trimethoprim-sulphonamide (SXT) resistance, while two clinical isolates did not. Additionally, four isolates from chicken manure and clinical children possessed tetracycline (TET) resistance.

**Figure 1:**
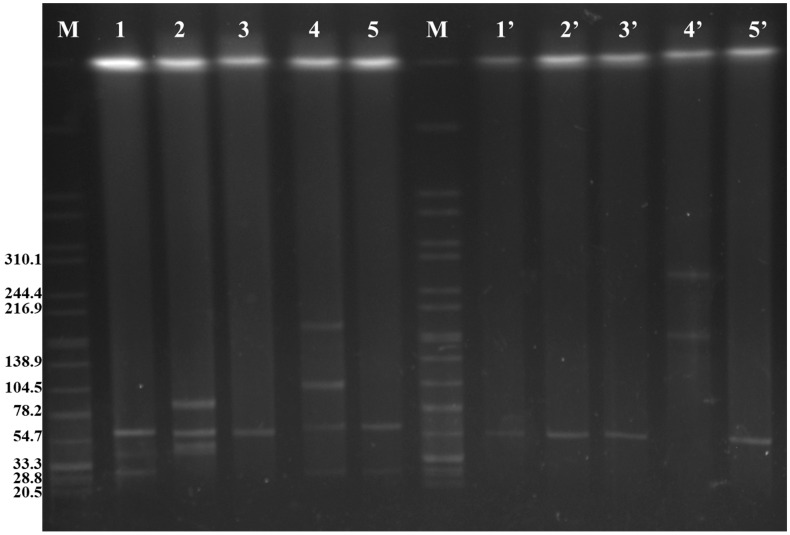
S1 nuclease pulsed-field gel electrophoresis of FosR *S.* Enteritidis donors (1–5) and corresponding *E. coli* trans-conjugants (1′–5′). M: H9812; 1: SJTUF 10993 and SJTUF 10994; 2: SJTUF 10959 and SJTUF 10960; 3: SJTUF 11346; 4: SJTUF 11561; 5: SJTUF 11565, SJTUF 11642, and SJTUF 11653; 1′: trans-conjugants of SJTUF 10993 and SJTUF 10994; 2′: trans-conjugants of SJTUF 10959 and SJTUF 10960; 3′: trans-conjugants of SJTUF 11346; 4′: trans-conjugant of SJTUF 11561; and 5′: trans-conjugants of SJTUF 11565, SJTUF 11642, and SJTUF 11653.

A total of five types of Inc groups were detected in these *fosA3*-positive isolates, including Inc HI2 (1/9, 11.1%), Inc I1 (3/9, 33.3%), Inc X (8/9, 88.9%), Inc FIIs (9/9, 100%), and Inc FIB (9/9, 100%) (Table 2). SJTUF 11346 only contained the Inc FIB and FIIs plasmid types, whereas Inc HI2 only existed in SJTUF 11561. The rest of seven isolates were divided into two groups: five possessing Inc FIIs, FIB, and X and two possessing Inc FIIs, FIB, X, and I1. S1-PFGE showed five different plasmid profiles in the nine Fos^R^ isolates (Figure 1, lines 1–5). In general, strains with uniform S1-PFGE profiles possessed the same plasmid types (Figure 1, lines 1, 2, and 5), such as SJTUF 10993, SJTUF 10994, SJTUF 10959, SJTUF 10960, SJTUF 11565, SJTUF 11642, and SJTUF 11653. Moreover, the latter five strains with the same plasmid types (IncFIIs, FIB, and X) showed two distinct S1-PFGE profiles (Figure 1, lines 2 and 5).

### 3.3. Conjugation Experiments

Conjugation experiments were performed to confirm the transmissibility of fosfomycin resistance in the recipient *E. coli* strain in nine Fos^R^ isolates at a frequency of 10^−4^–10^−6^ per donor cell (Table 2). Fosfomycin MICs for all trans-conjugants were ≥512 μg/mL. In eight of these Fos^R^ isolates, a single plasmid with the size of ~60 kb was transferred to the recipients (Figure 1, Lines 1′–3′ and 5′); moreover, two F-type plasmids (Inc FIIs and FIB) and the *fosA3* gene were also tested in these trans-conjugants (Table 2). The results showed that the fosfomycin resistance of the eight *fosA3*-harboring isolates was successfully transferred to recipient cells (Table 2) and that the *fosA3* gene was probably located on the Inc F plasmids. Notably, the plasmid type of the SJTUF 11561 trans-conjugant was the same as that of the parental isolate (Table 2). The S1-PFGE profile showed that two larger plasmids with sizes of ~150 kb and ~250 kb were detected in the trans-conjugant (Figure 1, line 4′). These results suggest that pairwise fusion likely occurred in the four tested plasmids (with sizes of ~30, ~60, ~120, and ~190 kb) of the parental strain SJTUF 11561. Unfortunately, these fusion plasmids were not sufficiently stable for further analysis.

### 3.4. Sequence Analysis of Plasmids in SJTUF 11561

Due to the co-occurrence of the *fosA3* gene and *bla*_CTX-M_ in SJTUF11561, it was selected for further sequencing. Additionally, this strain carried all four types of detected plasmids and had the highest transfer rate. According to WGS, five plasmids were identified in SJTUF 11561: p11561A, p115561B, p11561C, p11561D, and p11561E (Table 3). The molecular weights of the last four larger plasmids corresponded to the S1-PFGE analysis (Figure 1 and Table 3), whereas the smallest plasmid (p11561 A, ~8 kb) was not shown in the S1-PFGE pattern, possibly attributable to the display ability of the detection method. We confirmed that several antimicrobial resistance genes were located in three of these plasmids (p11561A, p115561B, and p11561C) (Table 3). Specifically, the *fosA3-, bla*_CTX-M-14_-, and *tetR*-bearing plasmid p11561A was 8056 bp in length but did not match the plasmid typing. Plasmid p11561B was 24,484 bp in size and belonged to the Inc X1 plasmid type, which harbors *aph(6)-Id*, *aph(3″)-Ib*, *bla*_TEM_, and *sul2* genes. Plasmid p11561C was 64,327 bp in size, comprising the backbone elements of the Inc FIB and Inc FIIs plasmids, indicating that p115561C is a hybrid plasmid bearing both virulence genes (*spvA, spvR, spvB,* and *pagC*) and the resistance gene *bla*_TEM_. Plasmids p11561D (109,062 bp, Inc I1-I) and p11561E (168,488 bp, Inc HI2) were not responsible for resistance genes. However, three virulence genes (*afaD*, *cia,* and *terC*) were present in the two plasmids (Table 3).

**Figure 2:**
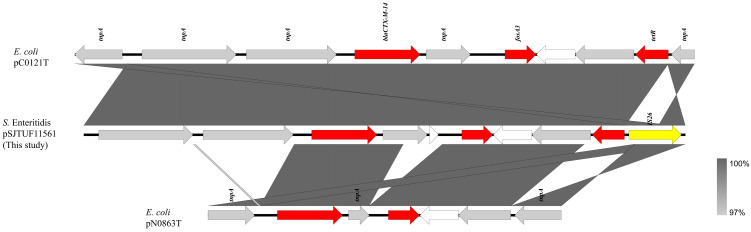
Genetic environment comparison of *fosA3* and *bla*_CTX-M-14_ genes in plasmids p11561A (this study), pC0121T (JX442753), and pN0863T (JQ823170). Gray shading indicates shared regions with a high degree of homology. Genes are represented by arrows and colored depending on gene function as depicted: red, antimicrobial resistance; yellow, mobile element; white, hypothetical protein; and gray, other protein (or genes).

The full-length fragment of pSJTUF11561(1–8056 bp) bearing *fosA3* and *bla*_CTX-M-14_ was compared with other plasmids identified using BLASTn. It showed similarity to a partial-length fragment of pC0121T (GenBank accession no. JX442753; 600–8000 bp; 91% coverage; 99.99% identity), which is an Inc FII resistance plasmid from *E. coli* (Figure 2). Sequence comparisons showed that all three resistance genes (*bla*_CTX-M-14_, *fosA3*, *tetR)* from both plasmids were surrounded by the transposase gene (*tnpA*); the difference was that IS*26* copies were inserted downstream of the resistance gene (*tetR)* in plasmid pSJTUF11561 but not in pC0121T. Moreover, the gene *tnpA* in the IS*26* copies was inverted, compared to that of pC0121T. The second plasmid pN0863T (accession no. JQ823170; 1–4731 bp; 50% coverage; 100.00% identity) showed a high similarity to pSJTUF11561 (Figure 2). Plasmid pN0863T was recovered from an *E. coli* isolated from the fecal specimens of a stray dog. The resistance gene *tetR* was not detected in pN0863T, and there was a pair of reversed *tnpA* genes at both ends of the selected fragment, in which the *tnpA* gene located downstream of the resistance gene (*fosA3*) was inverted, compared to the *tnpA* located on the IS*26* copies of plasmid pSJTUF11561. Moreover, between the two resistance genes (*bla*_CTX-M-14_ and *fosA3*) located in pSJTUF11561, there was more than one hypothetical protein when compared with those located in pC0121T and pN0863T.

## 4. Discussion

Previous studies have shown that the resistance rates of fosfomycin are significantly different among different Gram-negative bacteria isolated from clinical samples, being 9.6% (5/52) in *E. coli* [30], 36.1% (13/36) in *Pseudomonas aeruginosa* [30], 74.2% (23/31) in *Klebsiella pneumoniae* [30], 64.3% (9/14) in *Acinetobacter baumannii* [30], 9.5% (25/263) in *Shigella* [31], and 2.8% (14/501) in *S.* Enteritidis [16]. In the current study, the resistance rate of 119 strains of *S.* Enteritidis isolated from clinical children was 1.68% (2/119), which was significantly lower than the aforementioned Gram-negative bacteria. We speculate that there might be two possible reasons for this: (1) These selected isolates were from childhood cases, and fosfomycin is currently rarely used in children; therefore, it is less likely to acquire drug resistance, and (2) the current clinical application of fosfomycin is in combination with cephalosporins, aminoglycosides, quinolones, and other antibacterial drugs for the treatment of methicillin-resistant *Staphylococcus aureus* [32], MDR *Pseudomonas aeruginosa* (MDRP) [33], and *Shigella dysenteriae* [34], but the frequency of its use in the treatment of *Salmonella* infection is relatively low. In addition, compared with the recent reports by Gu et al. [11] and Fang et al. [12], in which the fosfomycin resistance rates of isolates from farm chickens and other food animals were 2.1% (6/288) and 2.6% (8/310), respectively, the resistance rate was a little higher (4.79%, 7/146) in retail chicken in this study, indicating the cumulative risk of fosfomycin resistance from broiler farming to retail. To the best of our knowledge, although Lin et al. [18] isolated two *fosA3*-harboring *Salmonella* isolates from retail chicken meat, this is the first report on the prevalence of fosfomycin resistance in *Salmonella* isolated from retail foods. We would like to expand the types of food and serotypes of *Salmonella* to enrich the survey data in future research.

FosA3 is the most frequently identified fosfomycin-modifying enzyme worldwide [4]. Inc FII plasmids play a predominant role in the dissemination of fosfomycin-modifying enzymes in Enterobacterales among clinical isolates and food animals in Asian countries [3,35,36]. Recent reports from China have described *fosA3* on transferable Inc FII plasmids in *S.* Enteritidis from chickens purchased from a market in Hong Kong [18] and from clinical isolates in Shanghai [16]. Similarly, the results from the plasmid typing of the parental isolate and its trans-conjugants in this study showed that the *fosA3* gene in eight Fos^R^ *S.* Enteritidis strains was also related to the Inc F-type plasmid.

The dissemination of the *fosA3* gene is closely associated with that of the ESBL gene *bla*_CTX-M_ [37,38,39]. Previous studies have reported that some plasmids from *Salmonella* carrying the *fosA3* gene also carried *bla*_CTX-M_ genes [17,40,41,42], whereas some *fosA3* genes in plasmids from *Salmonella* did not coexist with *bla*_CTX-M_ genes [12]. Both of the above situations were observed in this study; specifically, eight out of nine plasmids carried the *fosA3* gene, but no *bla*_CTX-M_ genes were detected (Table 2), whereas the *fosA3* gene was co-transferred with *bla*_CTX-M_ in the one remaining plasmid from SJTUF 11561 (Table 2). In previous studies, *bla*_CTX-M-14_ genes, which co-transferred with the *fosA3* gene in *Salmonella*, were usually detected in the Inc HI2 plasmid [12,16,19,43], and *bla*_CTX-M-55_ genes were detected in the Inc FII [12,16,18] or unknown-type plasmids [16]. Unlike previous studies, we found the coexistence of *fosA3* and *bla*_CTX-M-14_ genes on an 8 kb plasmid of unknown type (Figure 2), suggesting that plasmids associated with the co-transfer of *fosA3* and *bla*_CTX-M_ might be of diverse types.

A typical *fosA3* cassette (IS*26*-*fosA3*-*orf1*-*orf2*-*orf3*-IS*26*) can be randomly inserted into the area adjacent to the *bla*_CTX-M_ cassette in Enterobacterales to form different genetic structures [13,18,36,40]. In our study, the *fosA3* gene detected in the pSJTUF11561 plasmid was located in the genetic environment of IS*26*-IS*Ecp1*-IS*10*-*bla*_CTX-M-14_-ΔIS*903D*-*fosA3*-*orf1*-*orf2*-Δ*orf3*-IS*26*, which was also the typical *fosA3* cassette inserted downstream of a *bla*_CTX-M-14_ cassette (Figure 2). Sequence comparison analysis in this current study (Figure 2) showed that the genetic environment of *fosA3* of pSJTUF11561 was practically identical to two published plasmids isolated from *E. coli,* including pC0121T (Inc FII, IS*26*-IS*Ecp1*-IS*10*-*bla*_CTX-M-14_-ΔIS*903D*-*fosA3*-*orf1*-*orf2*-Δ*orf3*-IS*26*) [44] and pN0863T (Inc N, IS*26*-ΔIS*Ecp1*-*bla*_CTX-M-14_-ΔIS*903D*-*fosA3*-*orf1*-Δ*orf2*-IS*26*) [44]. It was surprising that the genetic environment of *fosA3* detected in pSJTUF11561 was different, with some plasmids in *Salmonella* carrying both *fosA3* and *bla*_CTX-M_. Specifically, the *fosA3* gene of *Salmonella* pGDD27–24 from a duck isolate had the common genetic background of IS*26*-*tetR*-*orf2*-*ofr1*-*fosA3*-IS*26*-*bla*_TEM-1_-*orf477*-*bla*_CTX-M-55_-ΔIS*Ecp1* [12], and the *fosA3* gene of *Salmonella* pYZU1189 from a healthy woman had the common genetic background of ΔIS*Ecp1*-*bla*_CTX-M-14_-IS*903B*-*fosA3*-*orf* [19]. Interestingly, the common genetic backgrounds of these two *fosA3* genes in these *Salmonella* strains were similar to that reported in *E. coli* [12,19]. These results suggest that plasmids carrying *fosA3* might be disseminated among Enterobacterales, not just among genera such as *Salmonella* or *E. coli*.

Furthermore, the S1-PFGE and PCR results of SJTUF 11561 showed that the number of plasmids decreased (Figure 1, lines 4 and 4′) and the size of plasmids increased (Figure 1, lines 4 and 4′), but the replication types did not change (Table 2) in the trans-conjugant, compared to the parental isolate, which revealed that the fusion occurred in the conjugation process. A similar phenomenon was reported previously, where both *fosA3* and *bla*_CTX-M_ genes located in *E. coli* plasmids isolated from chickens were successfully transferred to recipients by conjugation experiments, and the plasmid size in these trans-conjugants was much larger than that in the donor strains [45]. Multiple replicons and translocases were detected in the other four plasmids, which may be used to assist the transfer of *fosA3* and *bla*_CTX-M_ genes through the fusion process.

## 5. Conclusions

In summary, this is the first report on the prevalence rate of fosfomycin resistance in *Salmonella* Enteritidis isolated from retail chicken; moreover, this study demonstrated that Fos^R^ *Salmonella* Enteritidis can be detected in children with gastroenteritis who are currently rarely treated with fosfomycin. The plasmid-mediated *fosA3* gene played a predominant role in fosfomycin resistance because all of the FOS^R^ *Salmonella* Enteritidis isolates were positive for the *fosA3* gene and could be transferred by plasmids, including Inc F and the unknown type. Furthermore, the *fosA3* gene was co-distributed with other important antibiotic resistance genes, including *bla*_TEM_, *sul2*, *tetA*, and *bla*_CTX-M_. Our sequencing results support the idea that *fosA3* and *bla*_CTX-M_ might be disseminated among Enterobacterales but not limited to the *Salmonella* genus; therefore, further spread of these plasmids at the Enterobacterales level is a serious public health concern.

## Figures and Tables

**Table 1:** Minimal inhibitory concentration (MIC) of fosfomycin among 265 *Salmonella* Enteritidis isolates.

Origins of Strains (No.)	Distribution (No.) of MIC (μg/mL)							
	≤32	64	128	256	512	≥1024	Resistant Breakpoint	Resistance% (No.)
Retail chicken-related samples (146)	139	0	0	5	2	0	≥256	4.79% (7/146)
Children diarrhea samples (119)	117	0	0	2	0	0		1.68% (2/119)

**Table 2:** The antibiotic resistance phenotypes, resistance genes, and plasmid incompatibility group profiles of the fosfomycin-resistant strains.

Strains	Origins	MIC (μg/mL)	Antibiotics *	Genes Found	Plasmid Incompatibility (Inc.) Groups		Transfer Frequencies
					Parental	Tran-Conjugants	
SJTUF 11561	Chicken wings	512	AMP-CRO-STR-SUL-SXT-AZI-FOS	*fosA3, bla*_TEM_ *, sul2, bla*_CTX-M_	IncFIIs, Inc FIB, Inc HI2, Inc X, Inc I1	Inc FIIs, Inc FIB, Inc HI2, Inc X, Inc I1	10^−4^
SJTUF 10993	Chicken manure	512	AMP-STR-TET-SUL-SXT-FOS	*fosA3, bla*_TEM_ *, sul2, tetA*	Inc FIIs, Inc FIB, Inc X	Inc FIIs, Inc FIB	10^−5^
SJTUF 10994	Chicken manure	256	AMP-STR-TET-SUL-SXT-FOS	*fosA3, bla*_TEM_ *, sul2, tetA*	Inc FIIs, Inc FIB, Inc X	Inc FIIs, Inc FIB	10^−5^
SJTUF 11346	Chicken wings	256	SUL-FOS	*fosA3, sul2*	Inc FIIs, Inc FIB	Inc FIIs, Inc FIB	10^−5^
SJTUF 11565	Chicken heart	256	AMP-STR-SUL-SXT-FOS	*fosA3, bla*_TEM_ *, sul2*	Inc FIIs, Inc FIB, IncX	Inc FIIs, Inc FIB	10^−6^
SJTUF 11642	Chicken wings	256	AMP-STR-SUL-SXT-FOS	*fosA3, bla*_TEM_ *, sul2*	Inc FIIs, Inc FIB, IncX	Inc FIIs, Inc FIB	10^−6^
SJTUF 11653	Chicken liver	256	AMP-STR-SUL-SXT-FOS	*fosA3, bla*_TEM_ *, sul2*	Inc FIIs, Inc FIB, IncX	Inc FIIs, Inc FIB	10^−6^
SJTUF 10959	1-year-old boy	256	TET-SUL-FOS	*fosA3, sul2, tetA*	Inc FIIs, Inc FIB, Inc X, Inc I1	Inc FIIs, Inc FIB	10^−5^
SJTUF 10960	1-year-old boy	256	TET-SUL-FOS	*fosA3, sul2, tetA*	Inc FIIs, Inc FIB, Inc X, Inc I1	Inc FIIs, Inc FIB	10^−5^

* Tetracycline (TET), sulfisoxazole (SUL), ampicillin (AMP), ceftriaxone (CRO), streptomycin (STR), azithromycin (AZI), trimethoprim-sulfonamides (SXT), and fosfomycin (FOS).

**Table 3:** Plasmid analysis of SJTUF 11561 mined from whole genome sequencing.

Plasmid Names	Plasmid Sizes (bp)	Plasmid Types	Resistance Genes	Virulence Genes
p11561A	8056	\	*fosA3, bla*_CTX-M-14_ *, tetR*	\
p11561B	24,484	Inc X1	*aph(6)-Id, aph(3″)-Ib, bla*_TEM_ *, sul2*	\
p11561C	64,327	Inc FIB, Inc FIIs	*bla*_TEM_	*spvA, spvR, spvB, pagC*
p11561D	109,062	Inc I1-I	\	*afaD, cia*
p11561E	168,488	Inc HI2	\	*terC*

## Data Availability

The complete sequences of *S.* Enteritidis SJTUF11561 and its plasmids (p11561A, p115561B, p11561C, p11561D, and p11561E) have been deposited in the NCBI database under PRJNA1136444.

## Supplementary Materials

The following supporting information can be downloaded at: https://www.mdpi.com/article/10.3390/pathogens13090816/s1, Table S1. Primers used in PCR-based plasmid replicon typing; Table S2. Primers used for antimicrobial resistance genes.
