# Supplementary material for: Prevalence and Characteristics of Plasmid-Mediated Fosfomycin Resistance Gene fosA3 among Salmonella Enteritidis Isolates from Retail Chickens and Children with Gastroenteritis in China
Source: Pathogens. 2024 Sep 21;13(9):816. doi: 10.3390/pathogens13090816 (PMC11434640; doi:10.3390/pathogens13090816)
Supplement: Supplementary file 1 [file pathogens-13-00816-s001.zip › pathogens-3208263-supplementary.pdf]

Supplementary Table S1. Primers used in PCR assay[25]

**Primers**	**Location**	**Product size (bp)**	**Sequence**	**Annealing temperature (°C)**
Multiplex 1				
HI1	parA-parB	471	F- GGAGCGATGGATTACTTCAGTAC<br>R- TGCCGTTTCACCTCGTGAGTA	60
HI2	iterons	644	F- TTTCTCCTGAGTCACCTGTTAACAC<br>R- GGCTCACTACCGTTGTCATCCT	60
I1	RNAI	139	F- CGAAAGCCGGACGGCAGAA<br>R- TCGTCGTTCCGCCAAGTTCGT	60
Multiplex 2				
X	ori γ	376	F- AACCTTAGAGGCTATTTAAGTTGCTGAT<br>R-TGAGAGTCAATTTTTATCTCATGTTTTAGC	60
L/M	repA, B, C	785	F- GGATGAAAACTATCAGCATCTGAAG<br>R- CTGCAGGGGCGATTCTTTAGG	60
N	repA	559	F- GTCTAACGAGCTTACCGAAG<br>R- GTTTCAACTCTGCCAAGTTC	60
Multiplex 3				
FIA	iterons	462	F- CCATGCTGGTTCTAGAGAAGGTG<br>R- GTATATCCTTACTGGCTTCCGCAG	60
FIB	repA	702	F- GGAGTTCTGACACACGATTTTCTG<br>R- CTCCCGTCGCTTCAGGGCATT	60
W	repA	242	F- CCTAAGAACAACAAAGCCCCCG<br>R- GGTGCGCGGCATAGAACCGT	60
Multiplex 4				
Y	repA	765	F- AATTCAAACAACACTGTGCAGCCTG<br>R- GCGAGAATGGACGATTACAAAACTTT	60
P	iterons	534	F- CTATGGCCCTGCAAACGCGCCAGAAA<br>R- TCACGCGCCAGGGCGCAGCC	60
FIC	repA2	262	F- GTGAACTGGCAGATGAGGAAGG<br>R- TTCTCCTCGTCGCCAAACTAGAT	60

Multiplex 5				
A/C	repA	465	F- GAGAACCAAAGACAAAGACCTGGA<br>R- ACGACAAACCTGAATTGCCTCCTT	60
T	repA	750	F- TTGGCCTGTTTGTGCCTAAACCAT<br>R- CGTTGATTACACTTAGCTTTGGAC	60
FIIs	repA	270	F- CTGTCGTAAGCTGATGGC<br>R- CTCTGCCACAAACTTCAGC	60
singleplex 1				
F_{repB}	RNAI/repA	270	F- TGATCGTTTAAGGAATTTTG<br>R- GAAGATCAGTCACACCATCC	52
singleplex 2				
K	RNAI	160	F- GCGGTCCGGAAAGCCAGAAAAC<br>R- TCTTTCACGAGCCCGCCAAA	60
singleplex 3				
B/O	RNAI	159	F- GCGGTCCGGAAAGCCAGAAAAC<br>R- TCTGCGTTCCGCCAAGTTCGA	60

Supplementary Table S2. Primers used for antimicrobial resistance genes

**antibiotics**	**Target Gene**	**Primer Sequence**	**Annealing temperature (°C)**
fosfomycin	*fosA3*	F- GGCATTTTATCAGCAGT<br>R- AGACCATCCCCTTGTAG	54
	fosC2	F- CGAGCCAAGATTACTGT<br>R- AACGATTCCAAACGACT	54
	fosKP96	F- TATTAGCGAAGCCGATTTTGCT<br>R- CCCCTTATACGGCTGCTCG	54
β-lactams	bla_{TEM}	F- ATGAGTATTCAACATTTCCG<br>R- CTGACAGTTACCAATGCTTA	58
	bla_{CTX-M}	F- CGCTTTGCGATGTGCAG<br>R- ACCGCGATATCGTTGGT	52

	*bla*OXA	F- TCAACTTTCAAGATCGCA<br>R- GTGTGTTTAGAATGGTGA	49
tetracycline	*tetA*	F- GTAATTCTGAGCACTGTCGC<br>R- CTGCCTGGACAACATTGCTT	56
	tet B	F- CTCAGTATTCCAAGCCTTTG<br>R- ACTCCCCTGAGCTTGAGGGG	56
sulfonamides	*sul1*	F- CGGCGTGGGCTACCTGAACG<br>R- GCCGATCGCGTGAAGTTCCG	55
	sul2	F- GCGCTCAAGGCAGATGGCATT<br>R- GCGTTTGATACCGGCACCCGT	55
